# Can satellites and AI reveal the Maxwell equations of the Earth?

**DOI:** 10.1093/nsr/nwag210

**Published:** 2026-04-07

**Authors:** Lian Feng, Deren Li

**Affiliations:** State Key Laboratory of Information Engineering in Surveying, Mapping and Remote Sensing, Wuhan University, China; State Key Laboratory of Information Engineering in Surveying, Mapping and Remote Sensing, Wuhan University, China

The history of science is marked by rare yet transformative moments when scattered observations converged into concise, universal principles. Among them, Maxwell’s equations stand as a triumph of synthesis—unifying the once fragmented theories of electricity and magnetism through the interplay of empirical evidence, mathematical elegance, and theoretical imagination [1]. This unification not only led to the discovery of electromagnetic waves and laid the foundation of modern technology, but also profoundly deepened humanity’s understanding of nature while empowering the progress of modern civilization. Such breakthroughs were made possible by the steady accumulation of observations, the courage to challenge prevailing paradigms, and the relentless search for invariance amid apparent complexity.

In physics, the ultimate pursuit has been to distill the richness of the material world into a few governing equations. Earth system science now stands at a similar frontier: whether continuous, global observation can reveal an Earth analogue of Maxwell’s equations—not as a single, exact formulation in the classical sense, but as a modular family of interpretable, process-level governing relationships that organize behavior across scales and remain empirically testable within defined regimes.

Across the Earth system—spanning water and energy fluxes, carbon and nutrient cycling, and the exchanges of mass and momentum—numerous models and equations have been developed to describe individual processes. For example, Syukuro Manabe’s pioneering work demonstrated how rising atmospheric CO_2_ concentrations drive surface warming through physical modeling [2], laying the foundation for modern climate simulations and earning him the 2021 Nobel Prize in Physics. Yet in many other branches of Earth system science, existing models still stem largely from controlled laboratory experiments or limited field observations. In hydrology, for instance, empirical formulations often relate runoff or evapotranspiration to rainfall intensity, soil moisture, vegetation cover, and temperature [3]. However, their coefficients typically require site-specific calibration, limiting their transferability across regions and scales. This raises a fundamental question: can the behavior of natural processes, when viewed globally, also be captured by simple yet elegant mathematical expressions, revealing the hidden order beneath the apparent complexity of the Earth system?

**Figure 1.**
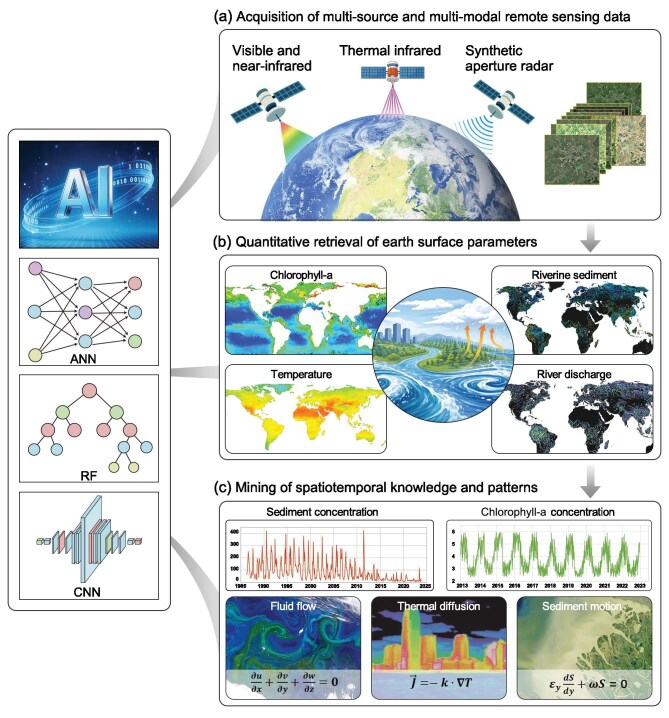
Satellites and AI as a planetary-scale laboratory for discovering governing relationships in the Earth system. (a) Global satellite observations provide continuous, multi-source, and multi-modal measurements of the Earth system across scales. (b) These observations are transformed into quantitative representations of key environmental variables through remote sensing retrieval and data integration. (c) By combining data-driven inference with physical constraints, spatiotemporal relationships can be distilled into a modular family of process-level equations and operators—an Earth-system analogue to Maxwell’s equations—that link subsystems across scales and remain empirically testable within defined regimes. ANN, artificial neural network; RF, random forest; CNN, convolutional neural network.

Bridging this gap requires continuous, globally consistent observations capable of capturing Earth’s processes in their natural variability and across diverse environmental settings. Modern Earth observation provides precisely this opportunity. Satellite and sensor constellations now enable temporally continuous measurements of the atmosphere, oceans, land surface, and cryosphere, while cameras with varying spatial resolutions and spectral sensitivities allow multiscale characterization of the planet’s dynamic state and its evolving trends across scales. Global satellite missions such as Landsat, MODIS, and Sentinel generate petabytes of data daily, while commercial constellations, including Planet’s fleet and the Oriental Star Constellation, are rapidly approaching daily, meter-scale global coverage through hundreds of optical and radar satellites (see Fig. 1). These vast datasets have already transformed environmental monitoring, supporting applications from mapping surface water and terrestrial vegetation to tracking oceanic phytoplankton dynamics [4–6].

This expansion of observation embodies the Fourth Paradigm of scientific discovery—data-intensive science [7,8]. Unlike traditional experimental, theoretical, or computational approaches, this new mode treats continuous, global data streams as both experiment and laboratory. Within these massive datasets may lie the fingerprints of universal order—patterns of conservation, balance, and interaction that reveal deep connections among processes once considered independent across scales. Yet, describing such complexity with simple equations remains a formidable challenge: the Earth system is inherently nonlinear, shaped by the intertwined flows of water, carbon, and energy, and increasingly modulated by human activity.

The rapid progress of artificial intelligence (AI), especially its ability to analyze and synthesize massive datasets, is now making this challenge increasingly within reach. AI has already become integral to remote sensing, enabling precise detection and interpretation of environmental parameters across all Earth system domains. The emergence of large-scale AI systems, such as Google’s AlphaEarth Foundation Model [9], illustrates how machine learning can integrate multisource satellite observations to enhance global environmental monitoring. Yet most current AI efforts remain focused on improving retrieval accuracy rather than uncovering the governing principles of Earth system behavior [10,11]. Increasingly, however, research is shifting toward this frontier: leveraging high-quality satellite data to identify process-level mechanisms and the interconnections that define Earth’s functioning.

Recent advances in data-driven modeling are extending this frontier by enabling the discovery of interpretable, transferable relationships directly from Earth system data [12,13]. Approaches

that integrate physical constraints with machine learning can infer explicit functional dependencies, enforce conservation principles, and generalize learned dynamics across spatial and temporal scales. Encouragingly, recent studies in hydrology, oceanography, and climate science demonstrate that such methods can recover relationships that are not only predictive, but also physically meaningful and transferable across regimes [14]. This shift also reflects an important distinction between conceptual paradigms and methodological tools: AI is used here as an umbrella term for learning-based computational systems, machine learning refers to specific algorithmic implementations, and data-driven modeling denotes a scientific paradigm focused on inferring governing relationships directly from observations rather than prescribing them *a priori*.

At the core of this emerging paradigm—distinct from generic data-driven modeling and focused on Earth system processes constrained by physics and global observation—is the vision of a universal framework for Earth system processes. Much as Maxwell’s equations unified the once fragmented phenomena of electromagnetism, such a framework aspires to integrate the planet’s diverse physical dynamics within a coherent, modular architecture. Each subsystem—whether governing the movement of water, the flow of energy, or the cycling of carbon—could, in principle, be expressed through interpretable, data-driven equations that capture their underlying dynamics. Within this framework, human processes need not be represented as continuous or conservative variables in the classical physical sense. Instead, they can be incorporated as external forcings, regime-switching operators, policy-driven boundary conditions, or learned adaptive components—explicitly capturing non-stationarity, institutional discontinuities, and strategic decision-making that are intrinsic to human systems. These data-derived formulations would not replace mechanistic theory but extend it, highlighting emergent constraints, dominant interactions, and scale-dependent regularities that govern system behavior. In doing so, they could illuminate how local disturbances propagate through interconnected networks, how feedbacks dampen or amplify variability, and how human activities reconfigure the stability of natural processes.

The implications of developing such a unified framework extend across both science and policy. Theoretically, it offers a means to view the Earth system as a single, integrated entity governed by consistent rules and symmetries. Practically, such equations could underpin predictive frameworks across disciplines: satellite-derived formulations of hydrological exchange may refine flood forecasting and water management; representations of carbon and energy balance could inform climate mitigation and adaptation; and surface heat flux relationships may strengthen models of ocean–atmosphere coupling. By explicitly incorporating human processes alongside natural ones, this Earth system framework could provide a quantitative basis for sustainable governance and planetary stewardship. Moreover, it would enable seamless integration of diverse data streams—from remote sensing imagery and *in situ* monitoring to historical archives—into a consistent, multi-scale representation of Earth system behavior. Unlike conventional Earth system models, where AI is primarily used to emulate parametrizations or accelerate numerical solvers, the proposed framework emphasizes the discovery and reformulation of governing relationships themselves. Rather than fitting parameters within predefined equations, it seeks to identify transferable, interpretable dependencies that organize Earth system behavior across processes and scales.

From a broader philosophical standpoint, the global satellite network represents more than an observational infrastructure—*it is humanity’s first true planetary-scale laboratory*. Just as Maxwell’s equations revealed the unity of electric and magnetic forces, the synthesis of satellite observation, AI, and physical reasoning may uncover the hidden symmetries binding Earth’s interlinked systems. This framework does not seek a single, all-encompassing ‘equation of Earth’, but rather a coherent ensemble of data-derived laws that collectively reveal the organizing principles of the coupled natural–human system. Through continuous observation, advanced computation, and the integration of physical knowledge, Earth system science may soon establish a framework that bridges process-level mechanisms with planetary-scale insights—transforming not only how we understand the planet, but how we sustain it in the Anthropocene.

Within the proposed framework, a longstanding critique of remote sensing can be reconsidered. Satellite observations are inherently retrospective, documenting states and processes that have already transpired. While such retrospective information is often viewed as limited for prediction, within a framework aimed at discovering governing relationships, past dynamics become the essential basis for inference. By discovering governing equations directly from past dynamics, we convert observation into inference and hindsight into foresight. The inferred relationships enable prediction of future system trajectories under evolving environmental and societal conditions, thereby transforming monitoring itself into a form of modeling.

Ultimately, this perspective delineates a new frontier for Earth system science: a synthesis of observation, computation, and theory. By deriving interpretable, data-driven formulations for each essential Earth system component, and by capturing their couplings and feedbacks, scientists can construct a modular framework describing the evolving dynamics of the coupled Earth–human system. This approach—ambitious yet achievable—calls on the scientific community to turn the torrents of satellite and sensor data into a coherent framework of governing relationships, unveiling the underlying principles of planetary behavior and its interconnections. Advancing this vision, however, requires continuous refinement of remote sensing capabilities—whether through physically grounded retrievals or AI-augmented approaches—as robust data inputs remain the foundation of accurate and interpretable outcomes [15].

At the same time, we explicitly acknowledge that this framework faces challenges common to data-driven discovery more broadly. A central difficulty lies in distinguishing genuine physical causality from spurious statistical correlations, particularly in systems where multiple mechanisms can generate similar observational signatures. Physically constrained learning provides one important pathway toward this goal by restricting solution spaces using conservation laws, process hierarchies, and cross-scale consistency, thereby reducing the risk of converging on statistically stable yet physically implausible relationships. Importantly, such approaches must explicitly account for uncertainty arising from incomplete observations and imperfect physical constraints. Any data-derived governing relationship should therefore be interpreted within clearly defined validity domains and rigorously evaluated across independent regimes, perturbations, and observational contexts.

In this sense, the search for Earth’s ‘Maxwell equations’ is not simply a pursuit of mathematical elegance, but a deeper scientific challenge—to reconcile observation with theory, to turn patterns into principles, and to transform an abundance of data into shared understanding.
